# The Risk Factors for Diabetic Retinopathy in a Chinese Population: A Cross-Sectional Study

**DOI:** 10.1155/2021/5340453

**Published:** 2021-01-28

**Authors:** Qingmin Sun, Yali Jing, Bingjie Zhang, Tianwei Gu, Ran Meng, Jie Sun, Dalong Zhu, Yaping Wang

**Affiliations:** ^1^Department of Medical Genetics, Nanjing University School of Medicine, Nanjing 210093, China; ^2^Department of Pharmacy, Jiangsu Province Hospital of Chinese Medicine, Affiliated Hospital of Nanjing University of Chinese Medicine, Nanjing 210029, China; ^3^Department of Endocrinology, Drum Tower Hospital Affiliated to Nanjing University Medical School, No321 Zhongshan Road, Nanjing 210008, China; ^4^Jiangsu Key Laboratory of Molecular Medicine, Nanjing University, Nanjing 210093, China

## Abstract

**Aims:**

Epidemiological data on diabetic retinopathy (DR) in Chinese population is still rather scarce, and risk factors for diabetic retinopathy are inconsistent because of study designs, grading standards, and population samples.

**Materials and Methods:**

This hospital-based retrospective study included 1052 type 2 diabetes patients. Diabetic retinopathy was diagnosed by nonmydriatic fundus photography and/or fundus examination apparatus. Logistic regression analysis was performed to evaluate the risk of diabetic retinopathy.

**Results:**

A total of 352 (33.5% prevalence) subjects were diagnosed with diabetic retinopathy based on our population. The patients in the DR group not only had significantly higher hemoglobin A1c (HbA1c), fasting plasma glucose (FPG), urinary microalbumin-creatinine ratio (ACR), and systolic blood pressure but also had higher follicle-stimulating hormone (FSH), luteinizing hormone (LH), and sex hormone-binding globulin (SHBG) levels compared to those in the non-DR group. Moreover, we confirmed that diabetes duration and HbA1c are strongly associated with DR risk. We also found that serum LH was an independent risk factor in male diabetic retinopathy patients (OR = 1.086, 95% CI 1.024–1.152), and the levels of LH were significantly associated with diabetic retinopathy prevalence (*P* = 0.018).

**Conclusions:**

Our study strengthens the argument that diabetes duration and HbA1c are risk factors for patients with DR. Additionally; we firstly confirmed that serum LH was an independent risk factor in male diabetic retinopathy patients.

## 1. Introduction

Diabetic retinopathy (DR) is a common microvascular complication of diabetes and is the first leading cause of irreversible vision loss in people of working age. A global meta-analysis reported that nearly one-third of diabetic patients have been diagnosed with diabetic retinopathy [1]. Diabetic retinopathy has been considered to be correlated with a higher risk of systemic vascular complications, such as nephropathy, peripheral neuropathy, and cardiovascular events, all of which lead to poor quality of life [2]. Therefore, the study of related risk factors is conducive to predict the condition of diabetic retinopathy in clinic.

Several epidemiological studies have reported the risk factors of diabetic retinopathy and aiming at the prevention and management of the disease, including a series of cross-sectional studies or cohort studies [3–5]. However, epidemiological data on diabetic retinopathy in Chinese population is still rather scarce, and risk factors for diabetic retinopathy are inconsistent because of study designs, grading standards, and population samples. Previous studies had confirmed that a variety of risk factors are associated with the development of diabetic retinopathy, including the history of diabetes, glycosylated hemoglobin A1c (HbA1c) levels, hyperglycemia, dyslipidemia, hypertension, and obesity [6, 7]. Especially, the longer diabetes duration and higher levels of HbA1c have been recognized as the key risk factors for diabetic retinopathy in a global diabetic retinopathy study [1].

There is a growing body of evidence indicating that multiplicity and complexity of sex hormone action target organs, particularly in the diabetic setting [8]. Some evidence suggested sex hormones appear to play an important role in optic nerve pathologies and other eye diseases [9]. Moreover, recent findings showed women seem to be at a higher risk for diabetic macrovascular complications, but the consequences of microvascular complications may be greater in men [8]. Therefore, in our cross-sectional study, multiple regression analysis was used to investigate the independently risk factor of diabetic retinopathy either including gender, age, diabetes duration, hyperglycemia, body mass index (BMI), blood pressure, HbA1c, and sex hormones in patients of type 2 diabetes. In this study, we sought to explore the special risk factors associated with diabetic retinopathy in a Chinese population.

## 2. Materials and Methods

### 2.1. Subjects

Clinical data of 1052 patients with type 2 diabetes were collected retrospectively during a period between January 2016 and January 2018 in the department of endocrinology, Nanjing Drum Tower Hospital, including 724 males and 328 females, aged between 18 and 70 years. Diagnostic of type 2 diabetes were considered according to 2003 American Diabetes Association criteria [10]. International clinical diabetic retinopathy disease severity scale was adopted to grade the retinopathy [11]. Subjects who combined with acute complications of diabetes, serious infections and important viscera, organ (heart, liver, kidney, etc.) dysfunction, and malignant tumor were excluded. Patients who have taken drugs affecting sexual hormone levels in the past three months were also excluded. This study has been approved by the Ethics Committee of the Nanjing Drum Tower Hospital.

### 2.2. Demographic Data

Anthropometric data on the height, weight, body mass index (BMI), waist circumference, hip circumference, and waist to hip ratio (WHR) were obtained from each subject. BMI was estimated based on the formula: BMI = weight (kg)/height (m^2^) [2]. WHR was determined according to the ratio between the standing waist and hip circumference. Patients' diabetes duration, blood pressure, and smoking and drinking history were collected.

### 2.3. Biochemical Measurements

Biochemical measurements were performed after fasting for at least 10 hours. Fasting plasma glucose (FPG) was tested using a hexokinase method (TBA-200FR, Tokyo, Japan). Fasting plasma C-peptide (FCP), follicle-stimulating hormone (FSH), luteinizing hormone (LH), serum testosterone, sulfated dehydroepiandrosterone (DHEAS), and hormone-binding globulin (SHBG) were determined by chemiluminescence analysis (Siemens, Bad Nauheim, Germany). The method of high-pressure liquid chromatography is using for determining HbA1c. Triglycerides (TG), high-density lipoprotein cholesterol (HDL-c), low-density lipoprotein cholesterol (LDL-c), total cholesterol (TC), creatinine, and urea nitrogen were detected by automatic biochemical analyzer. The urine microalbumin-creatinine ratio (ACR) is determined by immunoturbidimetry.

### 2.4. Study Design

Based on international clinical diabetic retinopathy disease severity scale, the patients were divided into the diabetic retinopathy group (DR group) and nondiabetic retinopathy group (non-DR group) by nonmydriatic fundus photography and/or fundus examination apparatus. Patients in the diabetic retinopathy group are all nonproliferative diabetic retinopathy. We divided the diabetic retinopathy group into the mild nonproliferative diabetic retinopathy group (mild NPDR), moderate nonproliferative diabetic retinopathy group (moderate NPDR), and severe nonproliferative diabetic retinopathy group (severe NPDR) according to disease severity.

### 2.5. Statistical Analyses

All statistical analyses were performed using SPSS software, version 22.0 (SPSS Inc., Chicago, IL, USA). A value of *P* < 0.05 was considered statistically significant. Continuous variables are represented by mean ± standard deviation (SD), and categorical variables are represented by percentage. According to different types of variables, independent sample *t*-test, rank sum test (Mann–Whitney *U* test), or chi square test was used to compare outcomes between two independent groups. The Cochran-Armitage trend test was used to analyze the difference of incidence rate of diabetic retinopathy in different LH levels. Binary logistic regression analysis was performed to evaluate the risk of LH and diabetic retinopathy, adjusted for confounding factors including age, BMI, systolic blood pressure, diabetes durations, HbA1c, FPG, FCP, urinary ACR, FSH, LH, DHEAS, and SHBG, to clarify the relationship between serum LH level and diabetic retinopathy.

## 3. Results

### 3.1. Characteristics of Participants

A total of 1052 type 2 diabetes patients (328 females, 724 males) were included in this study, and 352 (33.5%, 352/1052) subjects were diagnosed with diabetic retinopathy (DR group), the other 700 (66.5%, 700/1052) patients without diabetic retinopathy (non-DR group). The mean ages and diabetes durations of the patients were 55.3 ± 9.8 years and 10.5 ± 6.4 years in the DR group and 52.7 ± 11.5 years and 7.1 ± 6.3 years in the non-DR group. As expected, the patients in the diabetic retinopathy group had significantly higher HbA1c (*P* < 0.001), FPG (*P* = 0.037), urinary ACR (*P* < 0.001), and systolic blood pressure (*P* = 0.001) compared to those in the non-DR group. Compared with the non-DR group, FCP was lower in the diabetic retinopathy group (*P* = 0.006). No difference was observed in BMI, WHR, smoking and drinking situation, TC, TG, HDL-c, LDL-c, BUN, and creatinine between the two groups. However, higher FSH (*P* < 0.001), LH (*P* = 0.001), and SHBG (*P* = 0.001) levels were observed in the diabetic retinopathy group. These results indicated that sex hormones may be associated with progress of diabetic retinopathy. All characteristics of subjects were summarized in Table 1.

### 3.2. Risk Factors for Diabetic Retinopathy

**Figure 1:**
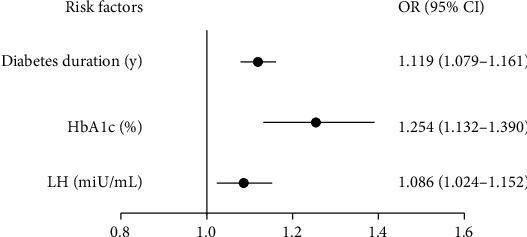
Independent risk factors for male diabetic retinopathy patients. Binary logistic regression analysis was performed by adjusting for age, smoking, drinking, systolic BP, BMI, HbA1c, TG, TC, FSH, T, DHEAS, and SHBG.

In regression analysis, diabetes duration (OR = 1.098, 95% CI 1.068–1.129) and HbA1c (OR = 1.195, 95% CI 1.103–1.295) were significantly associated with diabetic retinopathy for all the included subjects in this study. In further binary logistic regression analysis for males and females, diabetes duration (OR = 1.119, 95% CI 1.079–1.161), HbA1c (OR = 1.254, 95% CI 1.132–1.390), and LH (OR = 1.086, 95% CI 1.024–1.152) showed being independent risk factors for male diabetic retinopathy patients, after controlling for age, smoking, drinking, systolic BP, BMI, HbA1c, TG, TC, FSH, T, DHEAS, and SHBG (Figure 1). Interestingly, there was no risk association between the DR group and non-DR group in LH levels among female patients. Furthermore, we divide the female group into the premenopausal and postmenopausal groups; there was still no difference between the two groups. Additionally, according to the severity of diabetic retinopathy, we divided all the male diabetic retinopathy patients into three groups: the mild NPDR group, moderate NPDR group, and severe NPDR group.  Table 2 showed that only the duration of diabetes were positively associated with the degrees of diabetic retinopathy (*P* = 0.011), and no correlation between LH and diabetic retinopathy severity was found. Therefore, similar to previous studies, the present study confirmed that diabetes duration and HbA1c are strongly associated with diabetic retinopathy. Additionally, we also confirmed that serum LH was an independent risk factor in male diabetic retinopathy patients.

### 3.3. The Association of LH Levels and Diabetic Retinopathy Prevalence

**Figure 2:**
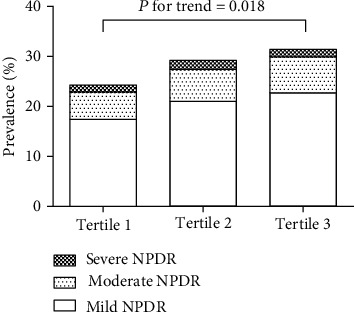
Prevalence of diabetic retinopathy by tertiles of LH. The Cochran-Armitage trend test was used to analyze the difference of incidence rate of diabetic retinopathy in different LH levels. With increasing levels of LH, there was a trend accompanied with higher prevalence of diabetic retinopathy.

Given that serum LH can have a role on male diabetic retinopathy, we further compared the levels of LH in the DR group and non-DR group in the male patients and found that the level of LH in the DR group was significantly higher (*P* < 0.001). Unexpectedly, a much less BMI was observed in the male DR group (*P* = 0.014). Similar to the results from all subjects, HbA1c, FPG, urinary ACR, and systolic blood pressure levels were also higher in the male DR group than those in the non-DR group (*P* < 0.050 for each) (Table 3). Furthermore, LH was divided into tertiles according to the expression levels in male diabetic retinopathy patients. We found that increasing levels of LH were accompanied with higher prevalence of diabetic retinopathy (*P* = 0.018, Figure 2). With increasing tertiles of LH, systolic BP, creatinine, and ACR was increased as well. Moreover, TC and LDL-c levels showed downtrends accompanied with LH tertiles (Table 4). These results showed that the levels of LH were significantly associated with diabetic retinopathy prevalence.

## 4. Discussion

In the present study, we found that 33.5% subjects were diagnosed with diabetic retinopathy in type 2 diabetes patients based on a Chinese population. The patients in the diabetic retinopathy group not only had significantly higher HbA1c, FPG, urinary ACR, and systolic blood pressure but also had higher FSH, LH, and SHBG levels compared to those in the non-DR group. Moreover, we confirmed that diabetes duration and HbA1c are strongly associated with diabetic retinopathy risk. Additionally; we firstly suggested that serum LH was an independent risk factor in male diabetic retinopathy patients, and the levels of LH were significant associated with diabetic retinopathy prevalence. It is suggested that LH can be used as a risk predictor of diabetic retinopathy in men.

Epidemiological data show that there are about 3.82 billion people diagnosed with diabetes globally at present, of which 1.26 billion people are suffering from diabetic retinopathy and about 370 million people have serious vision-threatening complications, which seriously affects the patient's daily activities and quality of life and overburdening the global health care system [12, 13]. Diabetic chronic microvascular complications mainly refer to vascular damage of the retina and kidney, which can also accelerate the occurrence of cardiovascular disease [14, 15]. Therefore, it is very important to find more risk factors for diabetic retinopathy. Previous studies have confirmed that the duration of diabetes, age, plasma glucose, blood lipid, blood pressure, HbA1c, and obesity are risk factors for diabetic retinopathy [7, 16]. Similarly, our results showed that compared with the non-DR group, the duration of diabetes in the diabetic retinopathy group was longer, and the systolic blood pressure, HbA1c, FPG, and urine ACR were significantly higher in the diabetic retinopathy group. The latest study showed that the concentrations of serum uric acid and urinary albumin are associated with the severity of DR in individuals with T2DM [17]. Moreover, our regression analysis also showed that diabetes duration and HbA1c were risk factors for diabetic retinopathy patients. Other risk factors of DR include nephropathy, dyslipidemia, smoking, and higher body mass index, which are revealed in previous studies [18]. Additionally, we firstly confirmed that serum LH was an independent risk factor in male diabetic retinopathy patients.

LH is included in the family of glycoprotein hormones. It is generated in the anterior pituitary gland and plays a very important role in gonad function [19]. As early as 1986, researchers detected LH exists in the vitreous humor of cadavers using radioimmunoassay. However, whether the quantification of LH in cadaveric eyes can reflect the physiological level of living eyes is still uncertain [20]. Until 1998, Thompson et al. discovered the expression of the luteinizing hormone receptor (LHR) gene in the neural retina and subsequently found that LHR was expressed in various organs such as the brain, placenta, skin, and kidney [10, 21]. The LHR transcription level in the retina is roughly equivalent to the LHR transcription level in the cerebral cortex. The density of LHR receptor transcripts and LHR protein is highest in retinal cone cells [21, 22]. It can be seen that the LH level in the eyes is correlated with the occurrence of retinal diseases. Movsas et al. collected vitreous samples from 40 adults (23 diabetics, 17 nondiabetics) and confirmed LH is present in the adult human eye. They also found that a diminution in LH receptor signaling negatively affects visual processing of the cone photoreceptors in adult mice [19]. Previous study showed that subjects with diabetic neuropathy had less testosterone and high LH and FSH levels [23]. In the present study, we detected that serum LH is an independent risk factor for male diabetic retinopathy patients. With the increasing of the LH level, the prevalence of diabetic retinopathy was increased as well.

As we all know, LH, the placental hormone, human chorionic gonadotropin, and the pituitary hormone can stimulate the same LH receptor (LHR) in the human body, including gonadal tissue and nongonadal organs containing the eyes. These hormones are known to promote vascular endothelial growth factor (VEGF) expression [22, 24]. Recently, a study found a strong correlation between LH and VEGF in mammalian eyes [25]. Previous studies demonstrated that VEGF dysregulation is a key factor in the pathogenesis of retinopathy of prematurity [26, 27] In vivo, researchers certified that LHR signaling has an important action in VEGF regulation and vascularization in the developing eye [28]. Therefore, the possible mechanisms that LH correlated to diabetic retinopathy could be explained by serum LH activating the LHR in the retina and induce VEGF expression, which leads to the occurrence of diabetic retinopathy. Certainly, the exact mechanisms between LH and diabetic retinopathy need to be further elucidated.

Several potential limitations of the present study should be clarified. Firstly, the current study is an observational study that needs long-term follow-up to support. Secondly, the study sample size is relatively small, especially in stratified analysis; subjects in the severe NPDR group are small, which may cause some bias although it does not affect the overall research results. Thirdly, this study lacks patients' eye samples to detect the LH and LHR expressions in eyes. The main reason is that none of the patients included in this study need surgical treatment. Lastly, our study only found correlation between LH and diabetic retinopathy incidence in men, which may depend on the sex hormone differences. Large sample study is still needed to further certify the phenotype. Therefore, we will continue to follow up these patients.

In summary, our present study confirmed that diabetes duration and HbA1c are strongly associated with diabetic retinopathy risk. Moreover, we firstly suggested that serum LH is significantly related to the occurrence of diabetic retinopathy in men, which provides a new starting point for predicting the incidence of the disease in male. Further studies with large sample and long-term follow-up are still needed to verify a more persuasive conclusion in all the population.

## Figures and Tables

**Table 1:** Characteristics of subjects with or without diabetic retinopathy.

	Non-DR	DR	*P* value
*n*	700	352	—
Age (y)	52.7 ± 11.5	55.3 ± 9.8	<0.001
BMI (kg/m^2^)	25.4 ± 3.8	25.0 ± 3.7	0.144
Waist circumference (cm)	91.7 ± 9.5	91.3 ± 9.8	0.568
Waist-to-hip ratio	0.9 ± 0.1	0.9 ± 0.1	0.275
Systolic BP (mmHg)	132.4 ± 16.4	136.5 ± 19.2	0.001
Diastolic BP (mmHg)	80.7 ± 11.8	81.0 ± 11.5	0.672
Smoking (%)	230 (32.9)	112 (31.8)	0.780
Drinking (%)	128 (18.3)	56 (15.9)	0.390
Diabetes duration (y)	7.1 ± 6.3	10.5 ± 6.4	<0.001
HbA1c (%)	8.7 ± 2.3	9.3 ± 2.2	<0.001
FPG (mmol/L)	8.4 ± 2.7	8.8 ± 3.1	0.037
FCP (pmol/L)	688.1 ± 371.1	617.7 ± 414.8	0.006
BUN (mmol/L)	6.2 ± 16.9	5.8 ± 3.1	0.711
Creatinine (*μ*mol/L)	60.9 ± 14.8	61.5 ± 15.2	0.488
ACR (mg/g)	42.0 ± 120.7	176.4 ± 538.2	<0.001
Triglycerides (mmol/L)	1.9 ± 2.4	1.8 ± 2.0	0.538
Total cholesterol (mmol/L)	5.0 ± 15.5	4.4 ± 1.2	0.485
HDL cholesterol (mmol/L)	1.3 ± 4.6	1.1 ± 0.3	0.493
LDL cholesterol (mmol/L)	2.6 ± 0.9	2.5 ± 0.9	0.425
FSH (mIU/mL)	18.1 ± 21.6	23.9 ± 25.2	<0.001
LH (mIU/mL)	9.0 ± 10.8	11.4 ± 10.6	0.001
Estradiol (pmol/L)	151.6 ± 125.2	153.2 ± 186.5	0.875
Testosterone (nmol/L)	8.5 ± 6.9	8.7 ± 10.9	0.770
DHEAS (*μ*g/dL)	165.1 ± 94.4	144.0 ± 80.7	0.006
SHBG (nmol/L)	30.8 ± 17.3	36.2 ± 19.9	0.001

Abbreviation: DR: diabetic retinopathy; BMI: body mass index; BP: blood pressure; FPG: fasting plasma glucose; FCP: fasting plasma C-peptide; BUN: blood urea nitrogen; ACR: urinary microalbumin-creatinine ratio; FSH: follicle-stimulating hormone; LH: luteinizing hormone; DHEAS: sulfated dehydroepiandrosterone; SHBG: sex hormone-binding globulin. Data are shown as mean ± SD or number (percentage).

**Table 2:** Characteristics of male subjects by the severity of diabetic retinopathy.

	Mild NPDR	Moderate NPDR	Severe NPDR	*P* value
*n*	169	51	13	—
Age (y)	54.8 ± 10.3	53.3 ± 8.8	50.7 ± 13.8	0.274
Systolic BP (mmHg)	135.2 ± 19.3	136.6 ± 16.9	135.5 ± 18.7	0.904
Diabetes duration (y)	9.9 ± 6.5	11.3 ± 5.4	15.1 ± 7.5^∗^	0.011
HbA1c (%)	9.2 ± 2.2	9.3 ± 2.0	9.3 ± 2.2	0.963
FPG (mmol/L)	8.7 ± 3.0	9.3 ± 4.1	8.1 ± 3.2	0.360
ACR (mg/g)	171.0 ± 538.7	295.4 ± 767.2	159.2 ± 167.8	0.428
LDL cholesterol (mmol/L)	2.4 ± 0.8	2.6 ± 0.9	2.3 ± 0.6	0.398
LH (mIU/mL)	6.3 ± 4.3	5.7 ± 3.0	6.1 ± 4.3	0.706

Abbreviation: NPDR: nonproliferative diabetic retinopathy; BP: blood pressure; ACR: urinary microalbumin-creatinine ratio; LH: luteinizing hormone. Data are shown as mean ± SD or number (percentage). ^∗^*P* < 0.05 versus the mild NPDR group; ^#^*P* < 0.05 versus the moderate NPDR group.

**Table 3:** Characteristics of male subjects with or without diabetic retinopathy.

	Non-DR	DR	*P* value
*n*	491	233	—
Age (y)	51.3 ± 11.7	54.3 ± 10.3	<0.001
BMI (kg/m^2^)	25.5 ± 3.7	24.8 ± 3.3	0.014
Waist circumference (cm)	92.9 ± 8.7	91.7 ± 8.9	0.141
Waist-to-hip ratio	0.9 ± 0.1	0.9 ± 0.1	0.991
Systolic BP (mmHg)	131.7 ± 15.6	135.6 ± 18.7	0.004
Diastolic BP (mmHg)	81.5 ± 11.5	81.9 ± 10.8	0.643
Smoking (%)	230 (46.8)	112 (48.1)	0.811
Drinking (%)	128 (26.1)	56 (24.0)	0.584
Diabetes duration (y)	6.7 ± 6.1	10.5 ± 6.4	<0.001
HbA1c (%)	8.7 ± 2.4	9.2 ± 2.2	0.003
FPG (mmol/L)	8.3 ± 2.7	8.8 ± 3.3	0.045
FCP (pmol/L)	698.1 ± 383.7	588.9 ± 309.8	<0.001
BUN (mmol/L)	6.5 ± 20.0	5.8 ± 1.4	0.605
Creatinine (*μ*mol/L)	66.2 ± 12.8	66.1 ± 13.2	0.958
ACR (mg/g)	37.3 ± 90.0	199.6 ± 587.8	<0.001
Triglycerides (mmol/L)	2.0 ± 2.8	1.9 ± 2.2	0.557
Total cholesterol (mmol/L)	4.3 ± 1.4	4.4 ± 1.3	0.772
HDL cholesterol (mmol/L)	1.3 ± 5.5	1.0 ± 0.3	0.525
LDL-cholesterol (mmol/L)	2.5 ± 0.9	2.5 ± 0.9	0.696
FSH (mIU/mL)	7.9 ± 6.3	10.5 ± 9.6	<0.001
LH (mIU/mL)	4.8 ± 3.3	6.1 ± 4.1	<0.001
Estradiol (pmol/L)	144.5 ± 49.8	158.7 ± 172.6	0.109
Testosterone (nmol/L)	12.1 ± 5.3	13.2 ± 11.5	0.177
DHEAS (*μ*g/dL)	189.5 ± 93.9	164.9 ± 80.4	0.009
SHBG (nmol/L)	28.8 ± 14.5	34.7 ± 17.2	<0.001

Abbreviation: DR: diabetic retinopathy; BMI: body mass index; BP: blood pressure; FPG: fasting plasma glucose; FCP: fasting plasma C-peptide; BUN: blood urea nitrogen; ACR: urinary microalbumin-creatinine ratio; FSH: follicle-stimulating hormone; LH: luteinizing hormone; DHEAS: sulfated dehydroepiandrosterone; SHBG: sex hormone-binding globulin. Data are shown as mean ± SD or number (percentage).

**Table 4:** Characteristics of male subjects by tertiles of LH.

	T1 (0.1-3.6)	T2 (3.6-5.38)	T3 (5.38-40.0)	P value
Age (y)	46.4 ± 11.5	52.8 ± 10.4^∗^	57.2 ± 9.3^∗^^#^	<0.001
BMI (kg/m^2^)	26.0 ± 4.5	25.4 ± 3.3	24.6 ± 2.7^∗^^#^	<0.001
Systolic BP (mmHg)	130.6 ± 17.0	132.4 ± 17.5	136.5 ± 16.3^∗^^#^	0.001
Diabetes duration (y)	5.1 ± 5.2	8.3 ± 6.1^∗^	10.1 ± 6.7^∗^^#^	<0.001
HbA1c (%)	9.2 ± 2.3	8.6 ± 2.2^∗^	8.9 ± 2.3	0.026
FPG (mmol/L)	8.9 ± 3.3	8.2 ± 2.5^∗^	8.4 ± 2.9	0.041
FCP (pmol/L)	707.1 ± 457.6	666.9 ± 321.2	627.3 ± 328.1	0.088
Creatinine (*μ*mol/L)	62.8 ± 11.5	66.4 ± 12.4^∗^	68.6 ± 14.1^∗^	<0.001
ACR (mg/g)	55.8 ± 203.4	53.3 ± 141.3	165.9 ± 564.6^∗^^#^	0.001
TC (mmol/L)	4.5 ± 1.3	4.3 ± 1.2^∗^	4.2 ± 1.1^∗^	0.021
LDL-c (mmol/L)	2.6 ± 0.9	2.5 ± 0.8	2.4 ± 0.9^∗^	0.032

Abbreviation: LH: luteinizing hormone; T1: tertile 1; T2: tertile 2; T3: tertile 3; BMI: body mass index; BP: blood pressure; FPG: fasting plasma glucose; FCP: fasting plasma C-peptide; ACR: urinary microalbumin-creatinine ratio; TC: total cholesterol; LDL-c, low-density lipoprotein cholesterol. Data are shown as mean ± SD or number (percentage). ^∗^*P* < 0.05 versus the T1 group; ^#^*P* < 0.05 versus the T2 group.

## Data Availability

The data used to support the findings of this study are available from the corresponding author upon request.

## References

[1] Yau J. W., Rogers S. L., Kawasaki R. (2012). Global prevalence and major risk factors of diabetic retinopathy. *Diabetes Care*.

[2] Liu Y., Yang J., Tao L. (2017). Risk factors of diabetic retinopathy and sight-threatening diabetic retinopathy: a cross-sectional study of 13 473 patients with type 2 diabetes mellitus in mainland China. *BMJ Open*.

[3] Zhang X., Saaddine J. B., Chou C. F. (2010). Prevalence of diabetic retinopathy in the United States, 2005-2008. *Journal of the American Medical Association*.

[4] Wang Y., Lin Z., Zhai G. (2020). Prevalence of and risk factors for diabetic retinopathy and diabetic macular edema in patients with early and late onset diabetes mellitus. *Ophthalmic Research*.

[5] Jammal H., Khader Y., Alkhatib S., Abujbara M., Alomari M., Ajlouni K. (2013). Diabetic retinopathy in patients with newly diagnosed type 2 diabetes mellitus in Jordan: prevalence and associated factors. *Journal of Diabetes*.

[6] Ting D. S., Cheung G. C., Wong T. Y. (2016). Diabetic retinopathy: global prevalence, major risk factors, screening practices and public health challenges: a review. *Clinical & Experimental Ophthalmology*.

[7] Kuo J. Z., Wong T. Y., Rotter J. I. (2014). Challenges in elucidating the genetics of diabetic retinopathy. *JAMA Ophthalmology*.

[8] Maricbilkan C. (2017). Sex differences in micro- and macro-vascular complications of diabetes mellitus. *Clinical Science.*.

[9] Nuzzi R., Scalabrin S., Becco A., Panzica G. (2019). Sex hormones and optic nerve disorders: a review. *Frontiers in Neuroscience*.

[10] Genuth S., Alberti K. G., Bennett P. (2003). Follow-up report on the diagnosis of diabetes mellitus. *Diabetes Care*.

[11] Wilkinson C. P., Ferris FL 3rd, Klein R. E. (2003). Proposed international clinical diabetic retinopathy and diabetic macular edema disease severity scales. *Ophthalmology*.

[12] Ogurtsova K., da Rocha Fernandes J. D., Huang Y. (2017). IDF diabetes atlas: global estimates for the prevalence of diabetes for 2015 and 2040. *Diabetes Research and Clinical Practice*.

[13] Gangwani R. A., Lian J. X., McGhee S. M., Wong D., Li K. K. (2016). Diabetic retinopathy screening: global and local perspective. *Hong Kong medical journal = Xianggang yi xue za zhi*.

[14] Grunwald J. E., Ying G. S., Maguire M. (2012). Association between retinopathy and cardiovascular disease in patients with chronic kidney disease (from the Chronic Renal Insufficiency Cohort [CRIC] Study). *The American Journal of Cardiology*.

[15] Son J., Jang E., Kim M. (2011). Diabetic retinopathy is associated with subclinical atherosclerosis in newly diagnosed type 2 diabetes mellitus. *Diabetes Research and Clinical Practice*.

[16] Cunhavaz J., Ribeiro L., Lobo C. (2014). Phenotypes and biomarkers of diabetic retinopathy. *Progress in Retinal and Eye Research.*.

[17] Chen D., Sun X., Zhao X., Liu Y. (2020). Associations of serum uric acid and urinary albumin with the severity of diabetic retinopathy in individuals with type 2 diabetes. *BMC Ophthalmology*.

[18] Lin K. Y., Hsih W. H., Lin Y. B., Wen C. Y., Chang T. J. (2021). Update in the epidemiology, risk factors, screening, and treatment of diabetic retinopathy. *Journal of Diabetes Investigation*.

[19] Movsas T. Z., Wong K. Y., Ober M. D., Sigler R. E., Lei Z. M., Muthusamy A. (2018). Confirmation of luteinizing hormone (LH) in living human vitreous and the effect of LH receptor reduction on murine electroretinogram. *Neuroscience*.

[20] Chong A. P., Aw S. E. (1986). Postmortem endocrine levels in the vitreous humor. *Annals Academy of Medicine Singapore.*.

[21] Thompson D. A., Othman M. I., Lei Z. M. (1998). Localization of receptors for luteinizing hormone/chorionic gonadotropin in neural retina. *Life Sciences.*.

[22] Dukic-Stefanovic S., Walther J., Wosch S. (2012). Chorionic gonadotropin and its receptor are both expressed in human retina, possible implications in normal and pathological conditions. *PloS one*.

[23] Ali S. T., Shaikh R. N., Ashfaqsiddiqi N., Siddiqi P. Q. R. (2009). Serum and urinary levels of pituitary-gonadal hormones in insulin-dependent and non-insulin-dependent diabetic males with and without neuropathy. *Archives of Andrology*.

[24] Trau H. A., Davis J. S., Duffy D. M. (2015). Angiogenesis in the primate ovulatory follicle is stimulated by luteinizing hormone via prostaglandin E2. *Biology of Reproduction*.

[25] Movsas T. Z., Sigler R. E., Muthusamy A. (2018). Vitreous levels of luteinizing hormone and VEGF are strongly correlated in healthy mammalian eyes. *Current Eye Research*.

[26] Hartnett M. E. (2015). Pathophysiology and mechanisms of severe retinopathy of prematurity. *Ophthalmology*.

[27] Hartnett M. E., Penn J. S. (2012). Mechanisms and management of retinopathy of prematurity. *The New England Journal of Medicine*.

[28] Movsas T. Z., Sigler R. E., Muthusamy A. (2018). Elimination of signaling by the luteinizing hormone receptor reduces ocular VEGF and retinal vascularization during mouse eye development. *Current Eye Research*.

